# Clinical failure of an extended-pulsed fidaxomicin regimen associated with emergence of a *Clostridioides difficile* isolate with reduced fidaxomicin susceptibility in an elderly man

**DOI:** 10.1128/aac.00130-26

**Published:** 2026-05-04

**Authors:** Claire E. Kaple, Sarah N. Redmond, Jennifer L. Cadnum, Bryan Hausman, Munok Hwang, Hosoon Choi, Piyali Chatterjee, Chetan Jinadatha, Curtis J. Donskey

**Affiliations:** 1 Department of Molecular Biology and Microbiology, Case Western Reserve University School of Medicine 196211, Cleveland, Ohio, USA; 2 Department of Medicine, Case Western Reserve University School of Medicine 220786, Cleveland, Ohio, USA; 3 Research Service, Louis Stokes Cleveland VA Medical Center 20083, Cleveland, Ohio, USA; 4 Department of Research, Central Texas Veterans Healthcare System 525981 , Temple, Texas, USA; 5 Department of Medicine, Central Texas Veterans Healthcare System 525981 , Temple, Texas, USA; 6 Department of Medical Education, College of Medicine, Texas A&M University 12332, Bryan, Texas, USA; 7 Geriatric Research, Education, and Clinical Center, Louis Stokes Cleveland VA Medical Center 20083, Cleveland, Ohio, USA; Houston Methodist Hospital and Weill Cornell Medical College, Houston, Texas, USA

**Keywords:** fidaxomicin, *Clostridioides difficile*, resistance, clinical failure

## Abstract

The clinical significance of *Clostridioides difficile* isolates with reduced fidaxomicin susceptibility is uncertain. We report treatment failure of extended-pulsed fidaxomicin treatment during every other day pulsing associated with emergence of a *C. difficile* isolate with reduced fidaxomicin susceptibility and a novel *rpoC* gene mutation, resulting in a novel amino acid substitution (R89K). The reduced susceptibility isolate had no reduction in growth or ability to colonize mice, but *in vitro* sporulation and toxin production were reduced.

## INTRODUCTION

Fidaxomicin is a macrocyclic antibiotic recommended as the preferred antimicrobial treatment for *Clostridioides difficile* infection (CDI) when available (1, 2). Fidaxomicin is as effective as oral vancomycin in achieving clinical cure but with a lower rate of recurrence (1, 2). The standard fidaxomicin regimen is 200 mg twice daily for 10 days (1, 2). An alternative extended-pulsed fidaxomicin regimen is 200 mg twice daily for 5 days, then once every other day for 20 days. In a randomized trial, the extended-pulsed fidaxomicin regimen was superior to a 10-day oral vancomycin regimen in achieving sustained clinical cure, presumably because the longer treatment duration provides more time for recovery of the intestinal microbiota and clearance of spores (3). Here, we present the case of an elderly man with clinical failure of an extended-pulsed fidaxomicin treatment regimen associated with emergence of a *C. difficile* isolate with reduced fidaxomicin susceptibility.

## CASE PRESENTATION

**Fig 1:**
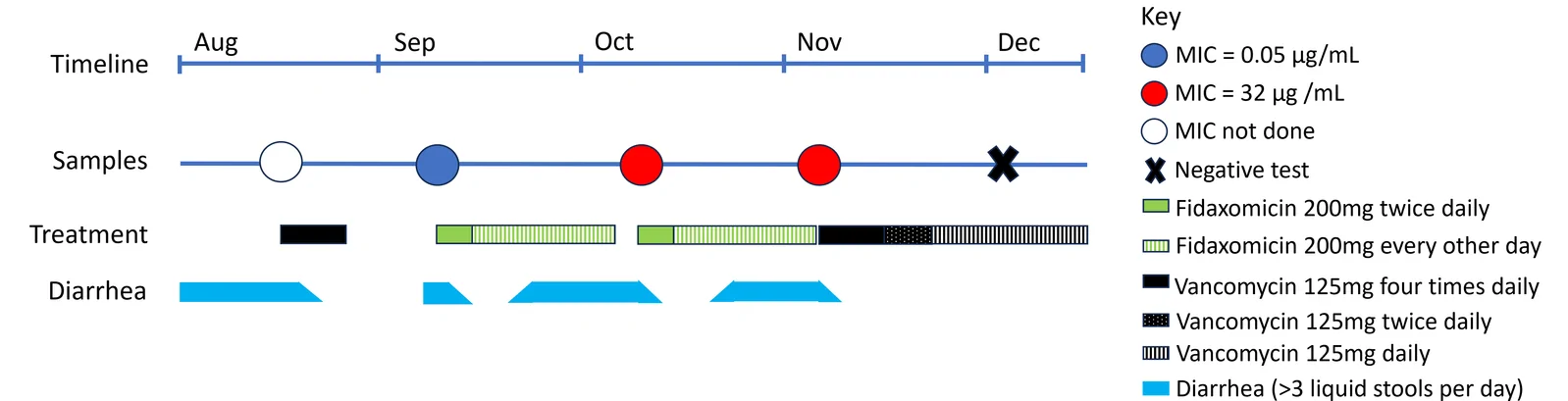
Timeline of diarrheal symptoms, *Clostridioides difficile* infection (CDI) treatments, and stool sample collection for CDI testing in an elderly man with clinical failure during two extended-pulsed fidaxomicin treatment regimens associated with emergence of a *C. difficile* isolate with reduced fidaxomicin susceptibility. The blue circle indicates a stool sample *C. difficile* isolate with fidaxomicin minimum inhibitory concentration (MIC) <2 µg/mL (i.e., fidaxomicin susceptible); the red circles indicate isolates with fidaxomicin MICs ≥ 2 µg/mL (i.e., reduced fidaxomicin susceptibility).

A 76-year-old man with emphysema was hospitalized with coronavirus disease 2019 and received treatment with azithromycin. He had no other antibiotic exposures within 90 days. Five days after completing azithromycin, he developed diarrhea with eight stools per day, and his stool tested positive for *C. difficile* by nucleic acid amplification test (NAAT) for genes that encode toxins and enzyme immunoassay (EIA) for toxins A and B. Figure 1 provides a timeline of diarrheal symptoms, CDI treatments, and stool sample collection. His initial CDI episode was treated with 10 days of oral vancomycin with resolution of symptoms, but diarrhea recurred 10 days later. His stool again tested positive by NAAT and EIA for toxins A and B. He received a fidaxomicin extended-pulsed regimen selected by his provider with resolution of diarrhea by day 5 of twice daily dosing, followed by recurrence during every-other-day dosing. His stool again tested positive by NAAT and EIA for toxins A and B. He received a second fidaxomicin extended-pulsed regimen and again experienced initial diarrhea resolution with recurrence during every-other-day dosing.

We determined fidaxomicin susceptibility of 3 of the patient’s *C. difficile* isolates using the reference agar dilution method (4). Reduced susceptibility was defined as MIC > 2 µg/mL (4). The initial isolate was fidaxomicin susceptible, with an MIC of 0.5 µg/mL, whereas the two subsequent isolates had fidaxomicin MICs of 32 µg/mL.

## MULTIPLE CHOICE QUESTION

Which statement is true regarding the management of CDI in a patient with clinical failure of fidaxomicin therapy?

- Clinical microbiology laboratories routinely culture stool specimens for *C. difficile* and test for susceptibility to fidaxomicin and vancomycin.
- Emergence of reduced fidaxomicin susceptibility during therapy has not been associated with treatment failure.
- For a third recurrence of CDI with an isolate with reduced fidaxomicin susceptibility, treatment with oral vancomycin and referral for fecal microbiota transplantation would be appropriate.
- Reduced susceptibility to fidaxomicin is due to constitutive expression of the *C. difficile vanG* operon.

Fidaxomicin targets bacterial RNA polymerase, resulting in inhibition of protein synthesis (4). *In vitro*, reduced fidaxomicin susceptibility has been associated with mutations in *rpoB* and *rpoC* genes that encode the RNA polymerase complex β and β′ subunits, respectively (5–7). Reduced fidaxomicin susceptibility has been rare in surveillance studies, but clinical *C. difficile* isolates with reduced susceptibility have been reported, often associated with mutations in *rpoB* or *rpoC* genes (6, 7). It is unclear if the emergence of reduced susceptibility occurs frequently because surveillance is limited and stool culture and susceptibility testing are not included in clinical diagnostic algorithms (4).

The clinical significance of reduced fidaxomicin susceptibility is uncertain. Fidaxomicin achieves high but widely variable concentrations in stool (mean, 1,225 ± 759, range 32 to 4,640 µg/g at the end of 10-day standard regimens) (8). Fidaxomicin extended-pulsed regimens could pose an increased risk of treatment failure if reduced susceptibility isolates emerge because stool concentrations will be relatively low during every-other-day dosing (9). Although some isolates with reduced susceptibility exhibit reduced growth, sporulation, and toxin production (5, 6), a *C. difficile* isolate with a *rpoB* mutation resulting in a fidaxomicin MIC of 128 μg/mL had similar *in vivo* virulence as the parental strain in mice (10). Moreover, Redmond et al. (4) recently reported emergence of *C. difficile* isolates with reduced fidaxomicin susceptibility associated with clinical failure in three patients receiving standard regimens. Based on these considerations, oral vancomycin is an appropriate therapeutic choice for CDI patients with clinical failure associated with emergence of isolates with reduced fidaxomicin susceptibility.

Answer: (C)

## TREATMENT STRATEGY AND OUTCOME

Management options were discussed with the patient and his family. The diarrheal episodes had caused substantial hardship because the patient was frail with limited mobility. The primary goal of the patient was to avoid future CDI episodes. Fecal microbiota transplantation was considered, but he was deemed to be at high risk for transplant failure due to frequent receipt of systemic antibiotics for emphysema exacerbations. The patient opted to receive a course of oral vancomycin, followed by lifelong daily suppressive vancomycin. His diarrhea resolved, and he had no further CDI episodes despite receiving courses of systemic antibiotics.

### Methods

The supplementary material provides details on the methods used to characterize the *C. difficile* isolates. Whole genome sequencing was used to identify mutations associated with reduced fidaxomicin susceptibility (4). Isolates were considered highly genetically related if they differed by ≤2 single nucleotide polymorphisms (SNPs) (4). A mouse model was used to assess the ability of *C. difficile* isolates to colonize mice pre-treated with clindamycin. *In vitro* growth rates, sporulation rates, and toxin production were measured (4). For the initial fidaxomicin-susceptible isolate and subsequent isolates with reduced susceptibility, linear mixed-effects models were used to compare *in vitro* growth, sporulation, and toxin production, and stool concentrations over time in colonized mice.

### Results

All three isolates belonged to multilocus sequence type 11 and were genomically related with 1–2 SNP differences (supplementary material). The reduced susceptibility isolates had G266A mutations in the *rpoC* gene coding for an R89K substitution in the RNA polymerase β′ subunit.

**Fig 2:**
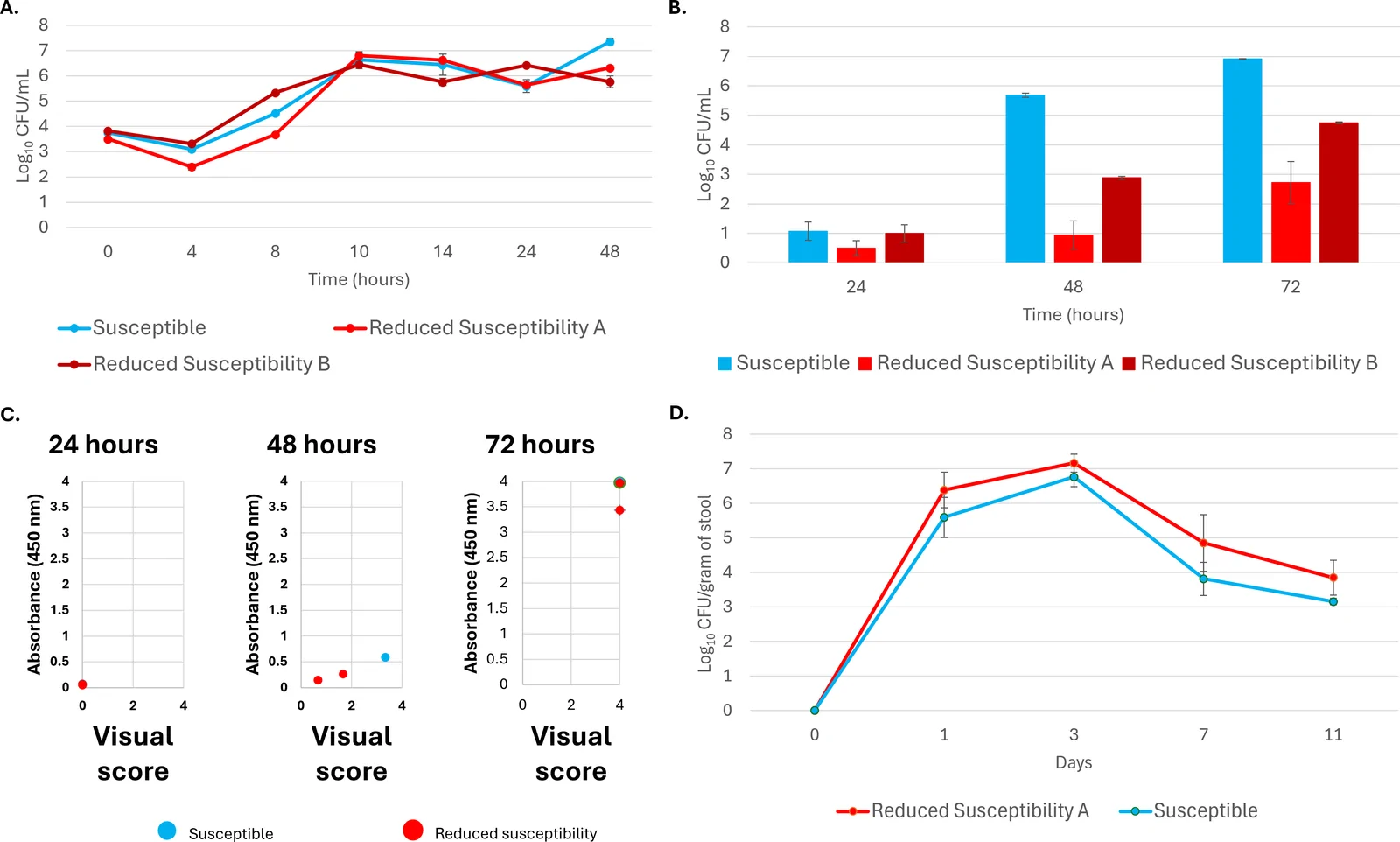
Comparison of *in vivo* growth rates (**A**), sporulation rates (**B**), and toxin production (**C**) for the fidaxomicin-susceptible isolate and two reduced susceptibility isolates from the patient with clinical failure during every other day dosing of two fidaxomicin extended-pulsed regimens, and *in vivo* colonization of mice pre-treated with subcutaneous clindamycin (**D**). Toxin production was estimated based on color intensity using a visual scale provided by the kit manufacturer (Supplemental material). CFU, colony-forming unit.

Figure 2 shows *in vitro* growth rates, sporulation rates, and toxin production, and *in vivo* stool concentrations in mice. In comparison to the susceptible isolate, the reduced susceptibility isolates demonstrated no difference in growth (*P* = 1) or in ability to colonize mice (*P* = 1), sporulation was significantly reduced (*P* < 0.01), but the isolates produced 2.7 to 4.8 log_10_ colony-forming units of spores by 72 h, and toxin production was reduced at 24 and 48 h (*P* < 0.001) but not at 72 h (*P* > 0.05).

### Discussion

To our knowledge, this is the first report of clinical failure of an extended-pulsed fidaxomicin regimen associated with emergence of reduced fidaxomicin susceptibility. The extended-pulsed regimen has been associated with a low risk for recurrence (3), but if reduced susceptibility isolates emerge, there may be an increased risk for treatment failure during every-other-day dosing in comparison to standard regimens. We hypothesize that the reduced susceptibility isolate that emerged caused recurrent diarrhea because it grew and produced toxin during periods when fidaxomicin levels in the colon were below the MIC.

Previous *in vitro* and clinical studies have demonstrated that mutations leading to R89G amino acid substitutions in the RNA polymerase β′ subunit result in reduced fidaxomicin susceptibility (7). Visualization of fidaxomicin-RNA polymerase interactions demonstrated that R89 interacts with fidaxomicin and the R89G amino acid substitution results in loss of a salt bridge interaction between β E1139 and β′ D237 (7). In our patient, a novel *rpoC* mutation and novel amino acid substitution at the same location (R89K) were associated with reduced fidaxomicin susceptibility. The isolates with reduced susceptibility demonstrated minimal alterations in fitness, with modest but significant reductions in sporulation and toxin production, but no reduction in growth or ability to colonize mice.

This case report and our similar report of clinical failure of standard fidaxomicin regimens (4) suggest that reduced susceptibility may be an underappreciated cause of fidaxomicin treatment failure. Practical approaches to routinely screen for reduced fidaxomicin susceptibility are needed.

## Data Availability

All genome sequences generated by this project have been submitted to NCBI (Bioproject ID PRJNA1199382). Reasonable requests for deidentified data will be honored by the corresponding author.

## SUPPLEMENTAL MATERIAL

Supplemental material aac.00130-26-s0001.docx Supplemental methods; Fig. S1.

ASM does not own the copyrights to Supplemental Material that may be linked to, or accessed through, an article. The authors have granted ASM a non-exclusive, world-wide license to publish the Supplemental Material files. Please contact the corresponding author directly for reuse.
