# Supplementary material for: Clinical failure of an extended-pulsed fidaxomicin regimen associated with emergence of a Clostridioides difficile isolate with reduced fidaxomicin susceptibility in an elderly man
Source: Antimicrob Agents Chemother. 2026 May 4;70(8):e00130-26. doi: 10.1128/aac.00130-26 (PMC13436074; doi:10.1128/aac.00130-26)
Supplement: Supplementary file 1 [file aac.00130-26-s0001.docx]

**Supplementary material**

**Clinical failure of an extended-pulsed fidaxomicin regimen associated with emergence of a *Clostridioides difficile* isolate with reduced fidaxomicin susceptibility in an elderly man**

**Susceptibility testing methods**

MICs for fidaxomicin were determined using the reference agar dilution method in accordance with CLSI recommendations [1] and as described by Thorpe et al. [2]. The test medium was brucella agar (Becton Dickinson, Sparksville, MD) supplemented with 5 mg/L hemin and 1 mg/L vitamin K1. Fidaxomicin (Tokyo Chemicals International, Tokyo, Japan) was dissolved in dimethyl sulfoxide (DMSO). Serial 2-fold dilutions of fidaxomicin were added to liquid agar in petri dishes and allowed to solidify. The test isolates were diluted to a 0.5 McFarland standard and inocula were deposited on the agar in a final concentration of 10^4^ CFU per spot. The plates were incubated anaerobically at 37°C with MICs read after 48 hours. The quality control strain was *C. difficile* American Type Culture Collection (ATCC) 700057. Reduced susceptibility (RS) was defined as MIC >2 µg/mL and resistance (R) was defined as MIC >16 µg/mL [3].

**References**

1). CLSI. Methods for Antimicrobial Susceptibility Testing of Anaerobic Bacteria. 9th ed. CLSI standard M11. Wayne, PA: Clinical and Laboratory Standards Institute; 2018.

2). Thorpe CM, McDermott LA, Tran MK, et al. 2019. U.S.-based national surveillance for fidaxomicin susceptibility of *Clostridioides difficile*-associated diarrheal isolates from 2013 to 2016. Antimicrob Agents Chemother 63:e00391-19.

3). Baines SD, Wilcox MH. 2015. Antimicrobial resistance and reduced susceptibility in *Clostridium difficile*: Potential consequences for induction, treatment, and recurrence of *C. difficile* infection. Antibiotics (Basel) 4:267-98.

**Methods for whole genome sequencing and bioinformatic analysis**

Whole genome sequencing was performed as previously described [1]. After extracting genomic DNA using genomic DNA purification kit (NEB, Ipswich, MA), libraries were prepared using Nextera DNA prep Kit (Illumina, San Diego, CA). Sequencing was performed on the NextSeq 550 sequencing system (Illumina, San Diego, CA). *De novo* assembly of reads was done using St. Petersburg genome assembler (SPAdes genome assembler (v.3.9)) on the Bionumerics calculation engine [2] and a multilocus sequence type (MLST) was assigned based on the PubMLST database [3]. The *rpoB and rpoC* genes were detected using BLAST v2.11.0 utilizing reference genes against the assembled sequences.

Trimmed reads were aligned to a *C. difficile* reference genome using Burrows-Wheeler Aligner-MEM v0.7.17 and variances were filtered using stringent filtering criteria. Positions with at least one unreliable (N) base, ambiguous (non-ACGT) base, or gap were removed. Non-informative SNP positions (i.e, positions that contain SNPs in comparison with the reference sequence, but all sample sequences have the same base) were also removed. Each retained SNP had a minimum 5x coverage and was covered at least once in the forward and reverse direction. If an entry did not have sufficient coverage at a specific SNP position, the SNP was removed for that entry, resulting in an empty cell in the SNP matrix. The SNP position was removed from the SNP matrix if none of the entries had sufficient coverage for a given SNP. The minimum distance between SNPs was 12 bp.

The reference sequence was strain S-0253 (Genbank GCA_018885085.1) for clade 1 isolates and strain R20291 (Genbank NC_013316.1) for clade 2 isolates. A dendrogram of the entire genome was created using Unweighted Pair-Group Method with Arithmetic averages (UPGMA) cluster analysis on Bionumerics software (v.8.1, Applied Maths). Isolates were considered genomically related if they differed by ≤2 single nucleotide polymorphisms (SNPs). All isolates will be deposited in NCBI.

**References**

1). Gonzalez-Orta M, Saldana C, Ng-Wong Y, Cadnum J, Jencson A, Jinadatha C, Donskey C. 2019. Are many patients diagnosed with healthcare-associated *Clostridioides difficile* infection colonized with the infecting strain on admission? Clin Infect Dis 30;69:1801-1804.

2). Bankevich A, Nurk S, Antipov D, et al. 2012. SPAdes: A new genome assembly algorithm and its applications to single-cell sequencing. J Comput Biol 19(5):455–77.

3). Jolley KA, Bray JE, Maiden MCJ. 2018. Open-access bacterial population genomics: BIGSdb software, the PubMLST.org website and their applications. Wellcome Open Res;3:124. doi: 10.12688/wellcomeopenres.14826.1.

Supplementary Figure Legend: Dendrogram showing the single nucleotide polymorphism (SNP) difference between the three isolates collected from the same patient. Isolate 1 differs from Isolate 2 and Isolate 3 by 2 SNPs and Isolate 2 and Isolate 3 differ from each other by 1 SNP

**Mouse model of *C. difficile* colonization**

The Animal Care Committee of the Cleveland VA Medical Center approved the experimental protocol. A mouse model of *C. difficile* colonization was used [1-3]. Female, 8-10 week old, Non-Swiss albino (CF-1) mice (8 per group) weighing ~30g (Inotiv, Indianapolis) were housed in individual cages with plastic filter tops during the experiments to prevent cross-contamination among animals. All mice received subcutaneous clindamycin 2 mg in 0.1 mL phosphate-buffered saline (PBS) once daily for 3 days to disrupt the microbiota that provide colonization resistance to *C. difficile* followed by oral gavage of 10^4^ CFU of *C. difficile* spores in 0.2 mL of sterile water. Stool pellets were collected at baseline and days 1, 3, 6, and 10 after inoculation of the fidaxomicin-susceptible and reduced susceptibility isolates.

*C. difficile* concentrations in stool specimens were quantified as described previously [1-3]. Stool specimens were emulsified in 10-fold (weight/volume) pre-reduced PBS. Serially diluted aliquots were inoculated onto pre-reduced cycloserine-cefoxitin-brucella agar containing 0.1% taurocholic acid and 5 mg/mL lysozyme (CDBA). The number of colony-forming units (CFU) per gram of stool was calculated. Linear mixed-effects models to compare stool concentrations over time in colonized mice.

**References**

1). Pearlmutter BS, Carlisle MG, Wilson BM, Sangwan N, Donskey CJ. 2024. Pulsed dosing and extended daily dosing of oral vancomycin do not facilitate clearance of *Clostridioides difficile* colonization in mice. Antimicrob Agents Chemother 68:e0090323.

2). Xu D, Mana TSC, Cadnum JL, Deshpande A, Afsari F, Sangwan N, Donskey CJ. 2022. Why Does Doxycycline Pose a Relatively Low Risk for Promotion of *Clostridioides difficile* Infection? Pathog Immun 2022;7:81-94.

3). Tomas ME, Mana TSC, Wilson BM, Nerandzic MM, Joussef-Piña S, Quiñones-Mateu ME, Donskey CJ. 2018. Tapering Courses of Oral Vancomycin Induce Persistent Disruption of the Microbiota That Provide Colonization Resistance to *Clostridium difficile* and Vancomycin-Resistant Enterococci in Mice. Antimicrob Agents Chemother 62:e02237-17.

**Evaluation of growth, sporulation, and toxin production**

We measured growth rates, sporulation rates, and toxin production in 2 strains with reduced fidaxomicin susceptibility (MIC 32 µg/mL) in comparison to the initial susceptible isolate (MIC 0.5 µg/mL) using previously reported methods [1]. Growth curves were performed over 24 hours in brucella broth with 5 mg/L hemin and 1 mg/L vitamin K1 [2]. Sporulation was assessed after 24, 48, and 72 hours of incubation. The log_10_ CFU of spores was measured after heat shock for 30 minutes at 65°C [ 3]. Toxin production was measured after 24, 48, and 72 hours of incubation using a toxin A/B enzyme-linked immunoassay (Techlab TOX A/B ELISA) with levels interpreted based on a visual score in accordance with the manufacturer’s package insert [4].

**References**

1). Redmond SN, Cadnum JL, Jencson AL, Kaple CE, Wilson BM, Skinner AM, Gargis AS, Hwang M, Choi H, Chatterjee P, Jinadatha C, Donskey CJ. Emergence and Spread of *Clostridioides difficile* Isolates With Reduced Fidaxomicin Susceptibility in an Acute Care Hospital. Clin Infect Dis. 2025 Jun 4;80(5):984-991.

2). Marchandin H, Anjou C, Poulen G, et al. In vivo emergence of a still uncommon

resistance to fidaxomicin in the urgent antimicrobial resistance threat *Clostridioides difficile*. J Antimicrob Chemother 2023; 78:1992–9.

3). Kuehne SA, Dempster AW, Collery MM, et al. Characterization of the impact of

rpoB mutations on the in vitro and in vivo competitive fitness of *Clostridium difficile* and susceptibility to fidaxomicin. J Antimicrob Chemother 2018; 73:973–80.

4). C. DIFFICILE TOX A/B II ™. TechLab. Available at: https://www.techlab.com/diagnostics/c-difficile/c-difficile-tox-ab-ii/
